# Uric acid transporters BCRP and MRP4 involved in chickens uric acid excretion

**DOI:** 10.1186/s12917-019-1886-9

**Published:** 2019-05-30

**Authors:** Xuedong Ding, Manman Li, Chenglu Peng, Zhi Wang, Shoufa Qian, Yuying Ma, Tianyi Fang, Shibin Feng, Yu Li, Xichun Wang, Jinchun Li, Jinjie Wu

**Affiliations:** College of Animal Science and Technology, Clinical Veterinary Medicine, Anhui Agricultural University, 130 West Changjiang Road, Hefei, 230036 China

**Keywords:** Uric acid, Breast cancer resistance protein, Multidrug resistance protein 4, Chickens

## Abstract

**Background:**

Breast cancer resistance protein (BCRP) and multidrug resistance protein 4 (MRP4) are involved in uric acid excretion in humans and mice. Despite evidence suggesting that renal proximal tubular epithelial cells participate in uric acid excretion in chickens, the roles of BCRP and MRP4 therein remain unclear. This study evaluated the relationship between BCRP and MRP4 expression and renal function in chickens.

**Results:**

Sixty laying hens were randomly divided into four treatment groups: a control group (NC) fed a basal diet; a sulfonamide-treated group (SD) fed the basal diet and supplemented with sulfamonomethoxine sodium via drinking water (8 mg/L); a fish meal group (FM) fed the basal diet supplemented with 16% fishmeal; and a uric acid injection group (IU) fed the basal diet and intraperitoneally injected with uric acid (250 mg/kg body weight). The results showed that serum uric acid, creatinine, and blood urea nitrogen levels were significantly higher in the SD and IU, but not FM, than in the NC groups. Renal tubular epithelial cells in the SD and IU groups were damaged. Liver BCRP and MRP4 mRNA and protein levels were significantly decreased in the SD and IU groups, but slightly increased in the FM group. In the SD group, BCRP and MRP4 were significantly increased in the ileum and slightly increased in the kidney. In the FM group, BCRP and MRP4 were significantly increased in the kidney and slightly increased in the ileum. In the IU group, BCRP and MRP4 were significantly increased in the kidney and ileum. BCRP and MRP4 expression in the jejunum was not affected by the treatments.

**Conclusion:**

Together, these results demonstrate that BCRP and MRP4 are involved in renal and intestinal uric acid excretion in chickens and that BCRP is positively related to MRP4 expression. Further, impairment of renal function results in an increase in serum uric acid as well as a compensatory increase in BCRP and MRP4 in the ileum; however, under normal renal function, renal BCRP and MRP4 are the main regulators of uric acid excretion.

## Background

Uric acid is the final product of purine metabolism. Dietary [1], genetic [2, 3], and disease-related [4] uric acid overproduction is the basis of hyperuricemia. However, the main cause of hyperuricemia is reduced uric acid excretion [5, 6]. In a study on uric acid metabolism in 65 patients with hyperuricemia, six patients (9.2%) exhibited an overproduction phenotype, 52 patients (80.0%) exhibited an underexcretion phenotype, and seven patients (10.8%) exhibited a mixed phenotype [5]. The kidney is the main organ responsible for uric acid excretion, accounting for approximately two-thirds of the total uric acid excretion in the body; the remaining one-third is mainly excreted via the intestines [7].

Uric acid excretion involves several uric acid transporters, such as breast cancer resistance protein (BCRP) [8], urate anion transporter 1 (URAT1) [9], multidrug resistance protein 4 (MRP4) [10], and organic anion transporters (OATs) [11]. BCRP and MRP4 are the major proteins involved in uric acid excretion. Previous studies have demonstrated that active uric acid secretion occurs in chicken renal proximal tubular epithelial cells (cPTCs) and that this may involve multiple uric acid transporters [12]. Bataille et al. [13] showed that in chickens, BCRP and MRP4 are expressed in cPTCs and that uric acid secretion is reduced by 60–70% in response to a 75% reduction in *MRP4* expression by short hairpin-mediated RNA interference. The net transepithelial transport of uric acid decreases when *BCRP* is knocked down [14], though the change is not significant, indicating that MRP4 is the main route for uric acid excretion in chicken proximal tubules.

BCRP is a high-capacity uric acid transporter that physiologically mediates renal and extra-renal (intestinal) uric acid excretion; its dysfunction leads to hyperuricemia [15]. Extensive data indicate that BCRP plays an important role in intestinal uric acid excretion in mice and humans [16–20]. Renal uric acid excretion is significantly reduced after nephrectomy in mice, whereas serum uric acid does not change and ileum BCRP expression is significantly increased [18]. Therefore, alterations in intestinal BCRP may serve as a compensatory mechanism. Similar to BCRP, MRP4 is a uric acid unidirectional efflux pump with multiple allosteric substrate-binding sites that is expressed in the apical membrane of human renal proximal tubules [21]. It is responsible for uric acid excretion by transporting uric acid from tubular epithelial cells into renal tubule lumens. MRP4 is also expressed in the basal membrane of human hepatocytes and is involved in the transport of uric acid in the liver [22]. In HEK293 cells, MRP4 can transport uric acid concurrently with adenosine monophosphate or guanosine monophosphate, and uric acid excretion increases upon overexpression of MRP4 [10].

Uricase in the mouse liver can convert uric acid into allantoin; however, human and chickens livers lack uricase [23]. Accordingly, the mechanism of uric acid metabolism in humans is different from that in mice. Therefore, chickens may constitute a more useful model than mice for studying human uric acid transporters. However, the roles of BCRP and MRP4 in uric acid excretion in chickens remain unclear. Therefore, this study aimed to investigate the relationship between serum uric acid levels and BCRP and MRP4 levels in the liver, kidney, and intestines, and to evaluate kidney and extrarenal uric acid excretion in chickens. Our findings may lay the foundation for the treatment and prevention of hyperuricemia.

## Methods

### Experimental design

Seventy 20-day-old Isa brown laying hens (weight, 189.3 ± 13.8 g) were purchased from Anhui Poultry Industry Co., Ltd. (China). Sixty healthy chickens were selected and were randomly divided into four treatment groups (*n* = 15 per group). The control group (NC) was fed a basal diet. The sulfonamide-treated group (SD) was fed the basal diet, and sulfamonomethoxine sodium soluble powder (Hengxin Pharmaceutical Co., Ltd., China) was added to the drinking water (8 mg/L). The fish meal group (FM) was fed the basal diet supplemented with 16% fishmeal (crude protein 27.6%) (defatted fish meal, China). The injection uric acid group (IU) was fed the basal diet and received uric acid (250 mg/kg body weight) via intraperitoneal injection every day; the uric acid (Sigma, USA) was suspended in 0.5% carboxymethyl cellulose-Na solution (Solarbio, China). All chickens were reared in cages at 25–30 °C and were allowed ad libitum access to feed and water. The basal diet was prepared based on nutritional requirements outlined by the National Research Council (1994) and contained 204.3 g/kg of crude protein, 11.5 g/kg of calcium, 4.2 g/kg of phosphorus, and 12.11 MJ/kg of metabolic energy. The experiment lasted for 3 weeks. At the end of the experiment (41 days of age), blood samples of 10 chickens from each group were collected from the jugular vein after a 12-h fasting. After clotting for approximately 30 min at room temperature, the blood was centrifuged at 3500×*g* for 10 min at 4 °C in a cryogenic centrifuge (TGL-18R, Hema, China) to obtain serum. The serum was stored at − 20 °C. Six chickens from each group were euthanized by decapitation. The liver, kidney, jejunum, and ileum were collected and divided into two portions, and then stored in 4% paraformaldehyde and liquid nitrogen, respectively. Renal cortex tissues were collected and fixed in 2.5% glutaraldehyde for transmission electron microscopy (TEM).

### Serum uric acid, creatinine, and blood urea nitrogen (BUN)

The amount of serum uric acid, creatinine, and BUN levels were determined in 10 samples per treatment group, as described previously [24], using an automatic biochemical analyzer (AU680, Beckman, USA). Serum uric acid was measured by a uricase method [25], creatinine was measured by a creatine oxidase method [26], and BUN was measured by a urease-glutamate dehydrogenase method [25].

### Transmission electron microscopy

TEM was was performed as previously described by Wang et al. [27], with some modifications. Renal cortex tissues from 3 chickens per group were collected for TEM. The tissue samples were fixed in 2.5% glutaraldehyde for 12 h (4 °C), followed by washing with phosphate buffer and fixing in 1% osmic acid for 2 h. The samples were dehydrated in a graded alcohol series (30, 50, 70, and 90%) and graded acetone (90 and 100%), and finally, the tissues were embedded in pure epoxy resin (EPON812, Serva FeinBiochemica, USA). Ultrathin sections (70 nm) were cut and were stained (uranyl and lead staining). Ultrastructural changes in renal tubular epithelial cells were observed under a transmission electron microscope (JEM-1230, Nippon Tekno, Japan) at 80.0 kv.

### Quantitative real-time PCR

Tissues from the liver, kidney, jejunum, and ileum (100 mg, *n* = 5 chickens for each group) were ground in liquid nitrogen and RNA was extracted using Trizol (Thermo Scientific, USA) based on published paper [28]. Next, 200 μl of chloroform was added to the sample, and then the sample was shaken 20 times, followed by centrifugation at 12,000 *g* for 15 min. Thereafter, 500 μl of the resulting supernatant was taken and then 500 μl of isopropanol was added followed by centrifugation at 12,000 *g* for 10 min. Next, the resulting supernatant was discarded and then 500 μl of 80% ethanol was added to the sample followed by centrifugation at 7500 *g* for 5 min, after which the supernatant was discarded. RNA concentrations were determined on a NanoVue Plus instrument (Thermo Scientific, USA). For each sample, 500 ng of total RNA was reverse transcribed (AT341, TransGen, China). QPCR was performed using an Arktik thermal cycler (Thermo Scientific, USA), and the cycle conditions were as follows: 2 min at 95 °C, 40 cycles of 15 s at 95 °C, 1 min at 60 °C, 30 s at 60 °C, 60–95 °C in 0.2 °C s^− 1^, and 10 s at 20 °C. Primers targeting chicken *BCRP* and *MRP4* are listed in Table 1.

**Table 1:** Sequence information of the primers used for QPCR

Gene	Primer Sequence (5′–3′)	Length	Accession No.
BCRP-F	CAGCAAGCAAGGAAGATCAC	129 bp	NM_001328490.1
BCRP-R	GGCTGGAGTTGAGATACTTC		
MRP4-F	TAGTGTTGGTCAGAGACAGC	167 bp	NM_001030819.1
MRP4-R	GTGCAATGGTCAGAACTGTG		
18S rRNA-F	CGGCGACGACCCATTCGAAC	99 bp	M_59389.1
18S rRNA-R	GAATCGAACCCTGATTCCCCGTC		

The QPCR data were analyzed using the 2^−ΔΔCt^ method. The mean threshold cycle value (Ct) of each sample was normalized to that of 18S rRNA. The mRNA relative expression levels were normalized to the average level of the NC group. The calculation procedures were as follows: −ΔCt = − (Target gene Ct - Reference gene Ct), 2^−ΔΔCt^ = 2^(−ΔCt(Target gene) - − ΔCt(control group gene))^. For *BCRP* gene, Ct values < 21.0 or > 26.0 were excluded; for *MRP4* gene, Ct values < 24.0 or > 29.0 were excluded; and for 18S rRNA gene, Ct values < 11.0 or > 16.0 were excluded.

### Western blotting

Total protein was extracted from the liver, kidney, jejunum, and ileum (100 mg, *n* = 3 chickens for each group) using RIPA cell lysis buffer (BL504A, Biosharp, China) and protein phosphatase inhibitor (P1260, Applygen, China). Protein concentrations were determined using a BCA protein concentration assay kit (BL521A, Biosharp, China). Proteins were electrophoresed using an electrophoresis apparatus (EPS 300, Tanon, China). The following antibodies were used: anti-BCRP (cat. no. bs-0662R, polyclonal, 1:1000, Bioss, China), anti-MRP4 (cat. no. bs-1422R, polyclonal, 1:1000, Bioss, China), anti-β-actin (cat. No. abs137975, monoclonal, 1:1000, Absin, China), and goat anti-rabbit IgG (cat. no. AP132P, 1:1000, Millipore, USA). Immunocomplexes were visualized using a western blotting detection kit (Advansta, USA) and blots were imaged using a ChemiDoc MP Imaging System (Bio-Rad, USA). Band densities were analyzed by Image-Pro Plus 6.0 and were normalized to those of β-actin. The protein relative expression levels were normalized to the average level of the NC group.

### Immunohistochemistry

Liver, kidney, jejunum, and ileum tissues were fixed in 4% paraformaldehyde, paraffin-embedded, and cut into 5-μm-thick sections. BCRP and MRP4 protein expression was detected by immunohistochemistry using anti-BCRP (1:400) and anti-MRP4 (1:300) antibodies, as described by Liu et al. [29]. Goat anti-rabbit IgG (1:1000) was used as the secondary antibody. The immunolabeled sections were observed and imaged under a light microscope (CX31, Olympus, Japan). Positive immunostained area and integrated optical density (IOD) were measured with Image-Pro Plus 6.0 [30]. Nine random images from 3 sections from each chicken were randomly selected for IOD analysis. For quantitative analysis of the results of immunohistochemistry, the average optical density was calculated as: IOD/ total area.

### Statistical analysis

The sample size was determined according to previous studies [16, 31, 32]. Data are expressed as the mean ± standard error (SE). Differences in serum parameters as well as BCRP and MRP4 levels between treatment groups and NC group were analyzed by one-way ANOVA using LSD and Duncan’s multiple comparison post test. A correlation analysis was conducted to explore the relationship between *BCPR* and *MRP4* by Pearson’s correlation coefficients. *P < 0.05* was regarded statistically significant. Statistical analyses were performed using SPSS Statistics (Version 25, IBM, USA). Graphs and scatter plots were generated using GraphPad Prism (version 5.01, GraphPad Software, USA).

## Results

### Serum uric acid, creatinine, and BUN levels

As shown in Table 2, serum uric acid levels were higher (*P = 0.01*) and creatinine and BUN levels were significantly higher (*P < 0.01*) in the SD group than in the NC group. Serum uric acid, creatinine, and BUN levels were significantly higher in the IU group than in the NC group (*P < 0.01*); however, there were no significant differences between the FM and NC groups.

**Table 2:** Determination of serum uric acid, creatinine, and BUN levels of chickens in different treatment groups

Groups	Uric (μmol/L)	Creatinine (μmol/L)	BUN (mmol/L)
NC	112.3 ± 4.8	2.79 ± 0.14	0.22 ± 0.01
SD	143.6 ± 17.7^*^	3.66 ± 0.24^**^	0.33 ± 0.03^**^
FM	113.8 ± 7.3	2.97 ± 0.15	0.22 ± 0.02
IU	156.6 ± 5.1^**^	3.79 ± 0.19^**^	0.28 ± 0.01^**^

^*^P < 0.05, ^**^P < 0.01 compared with the NC group

*NC*: control group; *SD*: sulfonamide-supplemented group; *FM*: fish meal group; *IU*: injection uric acid group. All data are means ± SE, *N* = 10 samples per treatment

### Ultrastructural analysis of renal tubular epithelial cells

**Fig. 1:**
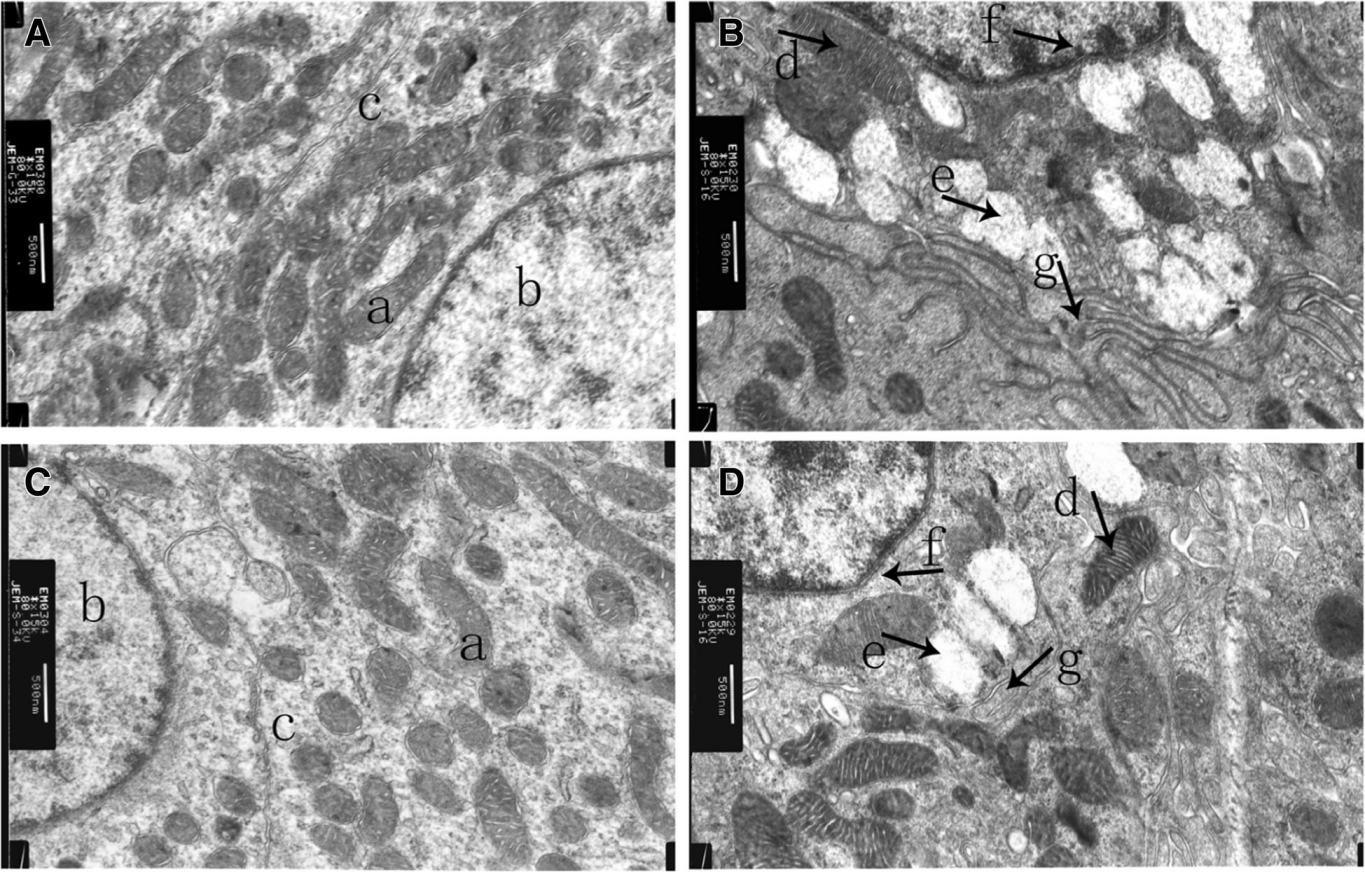
Ultrastructure of renal tubular epithelial cells. **A**: control group; **B**: sulfonamide-supplemented group; **C**: fish meal group; **D**: injection uric acid group. Photographs were taken by transmission electron microscope at 80.0 kV (15,000×). Scale bar = 500 nm. **a**: mitochondria; **b**: cell nucleus; **c**: endoplasmic reticulum; **d**: mitochondrial swelling; **e**: mitochondrial vacuolar degeneration; **f**: the nuclear membrane was irregular, and chromatin was condensed; **g**. endoplasmic reticular swelling

TEM revealed that the ultrastructure of renal tubular epithelial cells in the NC and FM groups (Fig. 1A, C) was intact; nuclei were round or oval, and the cytoplasm was uniform and abundant in mitochondria with regular, sharp ridges. In the SD and IU groups (Fig. 1B, D), nuclear membranes were irregular, the chromatin was condensed, some cells were apoptotic, the number of mitochondria was reduced, mitochondria were swollen, mitochondrial spinal were fractured or vague, and vacuolar degeneration in some mitochondria and endoplasmic reticular swelling were observed.

### BCRP and MRP4 mRNA and protein expression in normal control group chickens

**Fig. 2:**
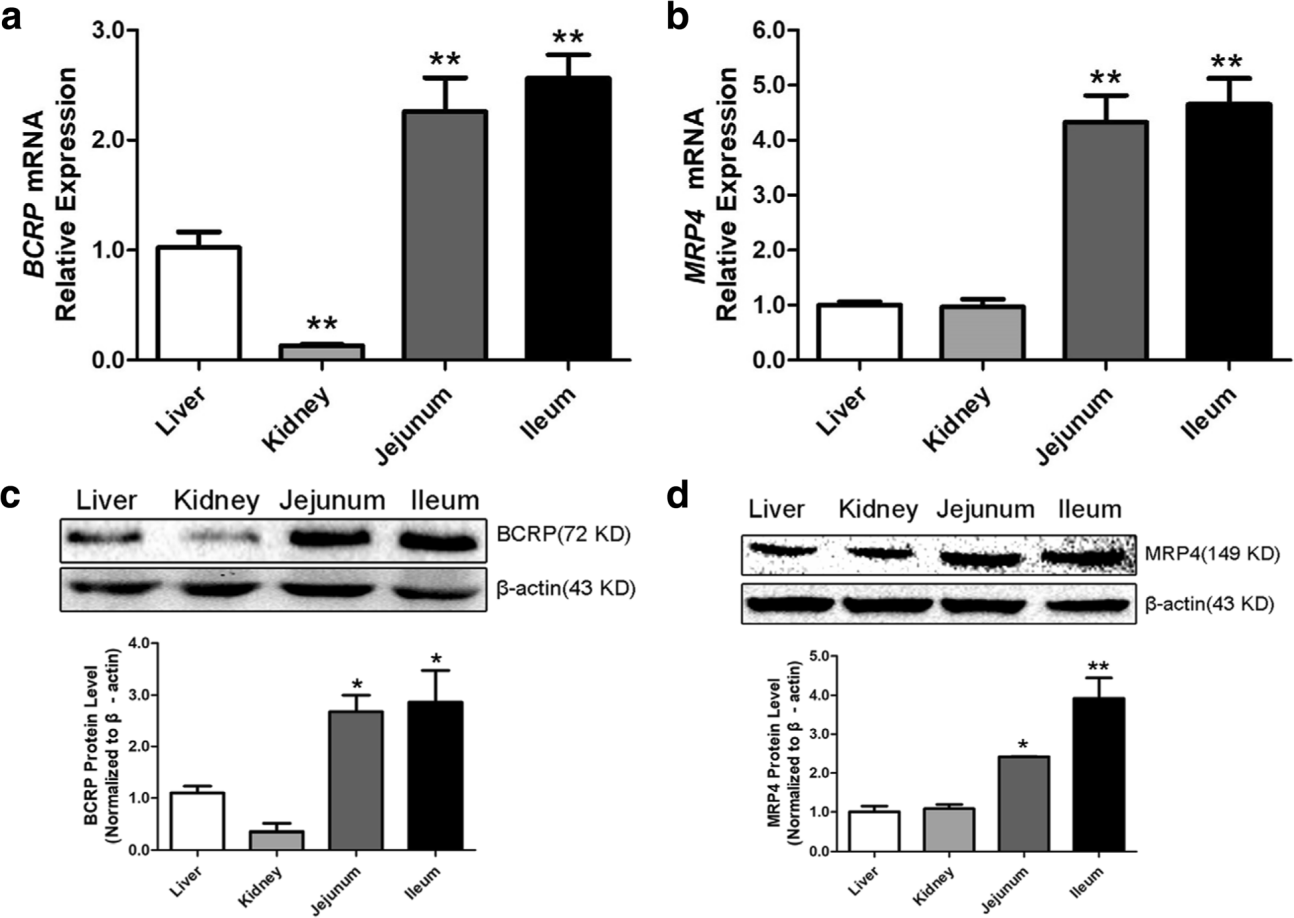
BCRP and MRP4 expression in the liver, kidney, jejunum, and ileum of control group chickens. **a** and **b**: *BCRP* and *MRP4* expression in the liver, kidneys, jejunum, and ileum of control group chickens (*N* = 5) analyzed by QPCR. ^*^P < 0.05, ^**^P < 0.01 compared with the liver. **c** and **d**: BCRP and MRP4 protein expression in the liver, kidneys, jejunum and ileum of control chickens by western blotting (*N* = 3). ^*^P < 0.05, ^**^P < 0.01 compared with the liver. All data are means ± SE. The mRNA and protein relative expression levels in kidney, jejunum and ileum were normalized to the average level of liver

**Fig. 3:**
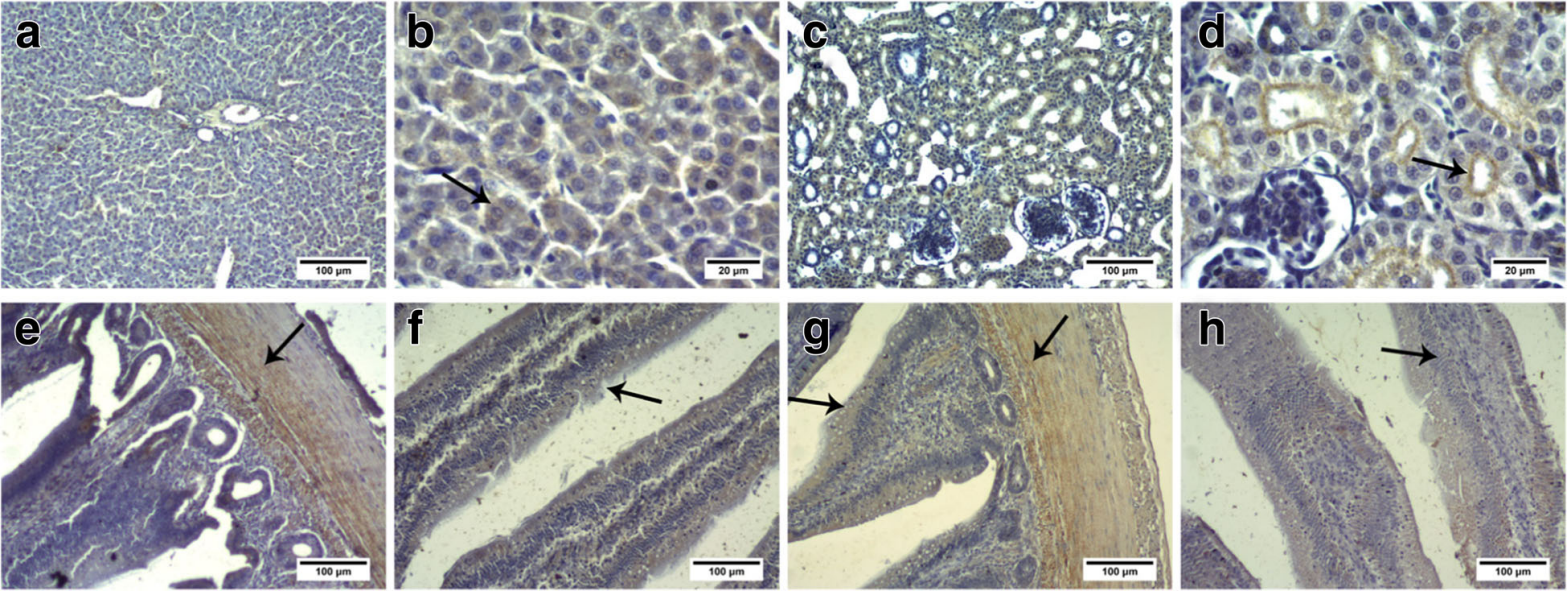
BCRP protein expression in the liver, kidney, jejunum, and ileum of control group chickens, as determined by immunohistochemistry. **a** and **b**: liver; **c** and **d**: kidneys; **e** and **f**: jejunum; **g** and **h**: ileum. **a**, **c**, **e**, **f**, **g**, and **h**: scale bar = 100 μm; **b** and **d**: scale bar = 20 μm. Immunohistochemical staining showed that BCRP protein was expressed in the liver cells, renal apical membrane, intestinal smooth muscle, and intestinal villi

**Fig. 4:**
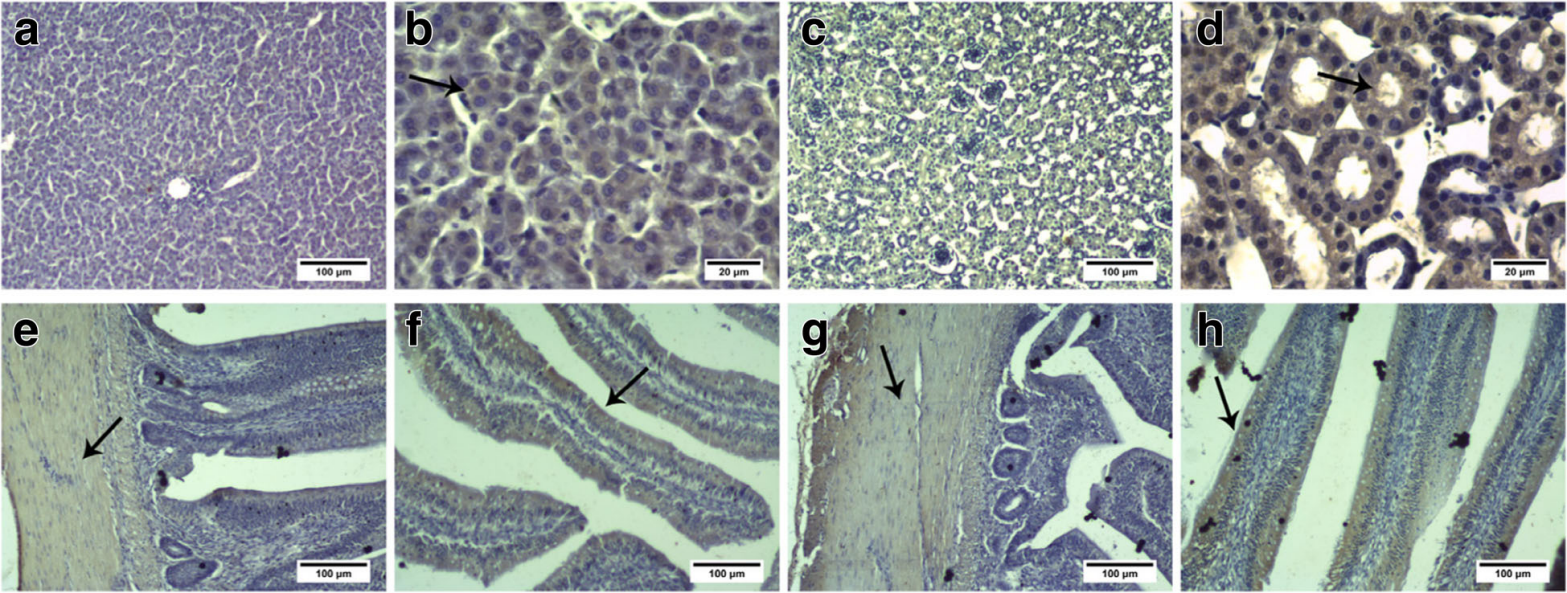
MRP4 protein expression in the liver, kidney, jejunum, and ileum of control group chickens, as determined by immunohistochemistry. **a** and **b**: liver; **c** and **d**: kidneys; **e** and **f**: jejunum; **g** and **h**: ileum. **a**, **c**, **e**, **f**, **g**, and **h**: scale bar = 100 μm; **b** and **d**: scale bar = 20 μm. Immunohistochemical staining showed that MRP4 protein was expressed in the liver cells, renal apical membrane, intestinal smooth muscle, and intestinal villi

QPCR and western blotting were used to detect the expression levels of BCRP and MRP4 in the liver, kidney, jejunum, and ileum of the NC chickens. BCRP (Fig. 2a, c) was highly expressed in the jejunum and ileum (*P* < 0.01), lowly expressed in the liver, and minimally expressed in the kidney (*P* < 0.01). MRP4 (Fig. 2b, d) expression levels were similar in the liver and kidney (*P* > 0.05) and were lower than those in the jejunum and ileum (*P* < 0.01). Immunohistochemical staining showed that BCRP and MRP4 were expressed in the liver cells, renal apical membrane, intestinal smooth muscle cells, and intestinal villi (Fig. 3 and Fig. 4).

### *BCRP* and *MRP4* expression in various treatment groups

**Fig. 5:**
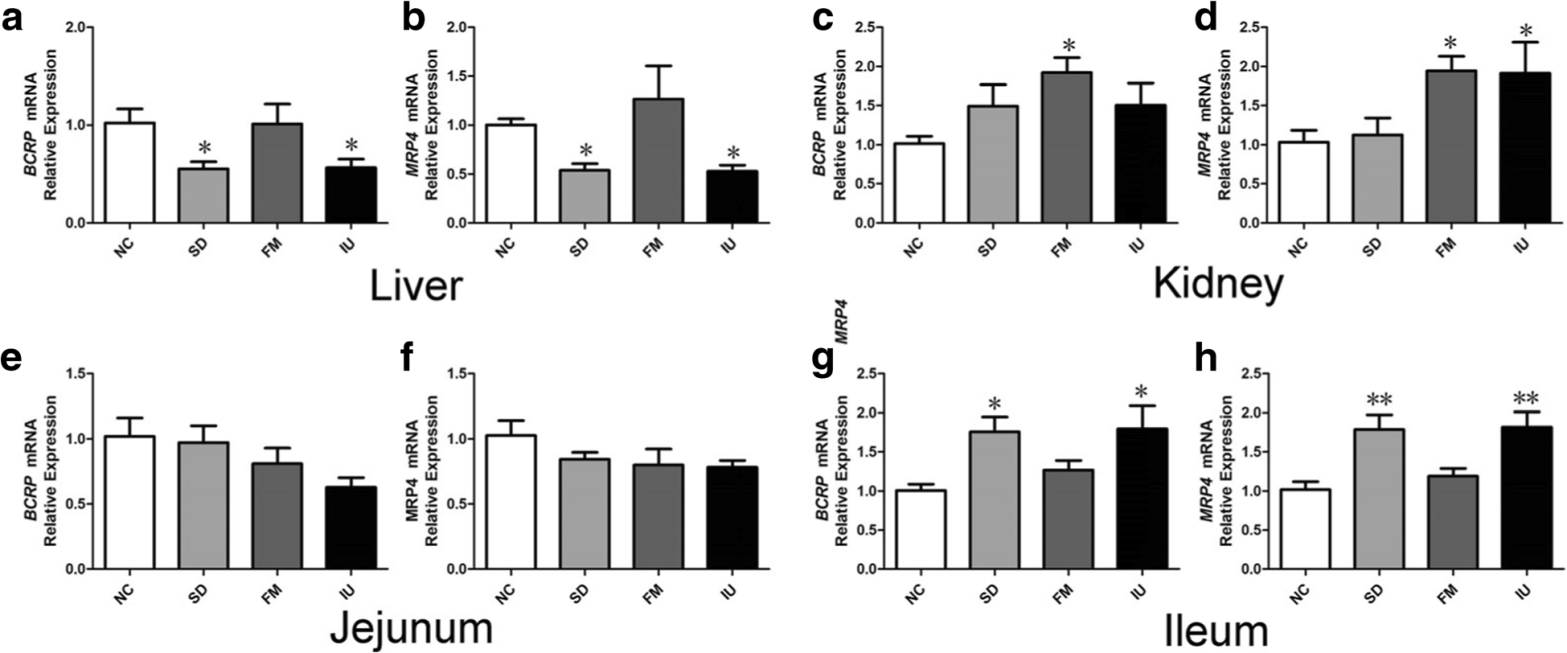
Relative *BCRP* and *MRP4* mRNA levels in the liver, kidney, jejunum, and ileum of chickens in different treatment groups, as determined by QPCR. NC: control group; SD: sulfonamide-supplemented group; FM: fish meal group; IU: injection uric acid group. **a** and **b**: *BCRP* and *MRP4* mRNA expression in the liver; **c** and **d**: *BCRP* and *MRP4* mRNA expression in the kidneys; **e** and **f**: *BCRP* and *MRP4* mRNA expression in the jejunum; **g** and **h**: *BCRP* and *MRP4* mRNA expression in the ileum. The mRNA relative expression levels were normalized to the average level of the control group. All data are means ± SE, ^*^P < 0.05, ^**^P < 0.01 compared with the NC group, *N* = 5 samples per treatment

*BCRP* and *MRP4* mRNA expression levels in the liver, kidney, jejunum, and ileum of chickens in various treatment groups were evaluated by QPCR. As shown in Fig. 5, *BCRP* and *MRP4* mRNA expression levels in the liver were significantly decreased in the SD and IU groups. However, the expressions of these genes in the kidney were increased in the FM group (*P* < 0.05). Renal *MRP4* mRNA expression was also significantly increased in the IU group as compared to that in the NC group (*P* < 0.05). Although *BCRP* mRNA level was slightly increased in the kidney, it was not significantly different from that in the NC group. Similar to their expression in the kidney, the expression of *BCRP* and *MRP4* in the ileum was significantly increased in the SD and IU groups (*P* < 0.05). In the FM group, renal *BCRP* and *MRP4* mRNA levels were significantly increased (*P* < 0.05 for both), ileal *BCRP* and *MRP4* mRNA levels were slightly increased, and liver *BCRP* and *MRP4* mRNA levels showed no obvious difference. *BCRP* and *MRP4* mRNA levels in the jejunum showed a decreasing trend in the three experimental groups, though the differences were not significant.

**Fig. 6:**
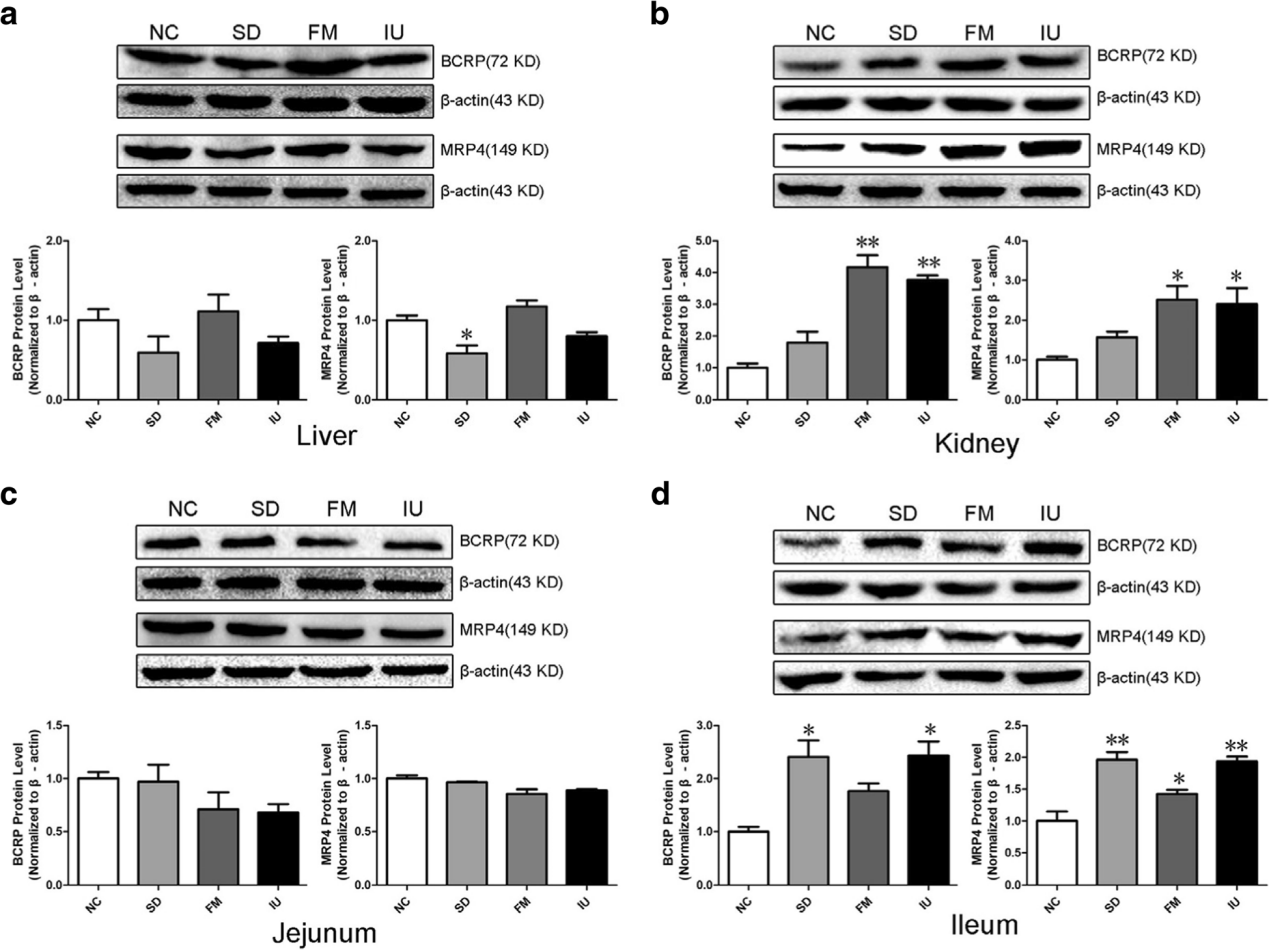
BCRP and MRP4 protein expression in the liver, kidney, jejunum, and ileum of chickens in different treatment groups, as determined by western blotting. NC: control group; SD: sulfonamide-supplemented group; FM: fish meal group; IU: injection uric acid group. **a**: Protein expression of BCRP and MRP4 in the liver; **b**: protein expression of BCRP and MRP4 in the kidney; **c**: protein expression of BCRP and MRP4 in the jejunum; **d**: protein expression of BCRP and MRP4 in the ileum. The protein relative expression levels were normalized to the average level of the control group. All data are means ± SE, ^*^P < 0.05, ^**^P < 0.01 compared with the NC group; *N* = 3 samples per treatment

Western blotting results showed that BCRP and MRP4 protein expression levels in the liver, kidney, jejunum, and ileum of each group were consistent with the mRNA expression levels (Fig. 6). In the SD and IU groups, liver BCRP and MRP4 levels were decreased, and the liver MRP4 level was significantly decreased in the SD group (*P* < 0.05). In the SD group, BCRP and MRP4 protein levels were slightly increased in the kidney and were significantly increased in the ileum (*P* < 0.05 and *P* < 0.01, respectively). In the IU group, BCRP and MRP4 protein levels were significantly increased in the kidney and ileum (*P* < 0.05 and *P* < 0.01, respectively). In the FM group, BCRP and MRP4 protein levels were significantly increased in the kidney (*P* < 0.01 and *P* < 0.05, respectively), ileal BCRP protein levels were slightly increased, and ileal MRP4 protein levels were significantly increased (*P* < 0.05), whereas there was no significant difference in the liver level compared to the control group. BCRP and MRP4 protein levels in the jejunum did not differ significantly in the three experimental groups.

**Fig. 7:**
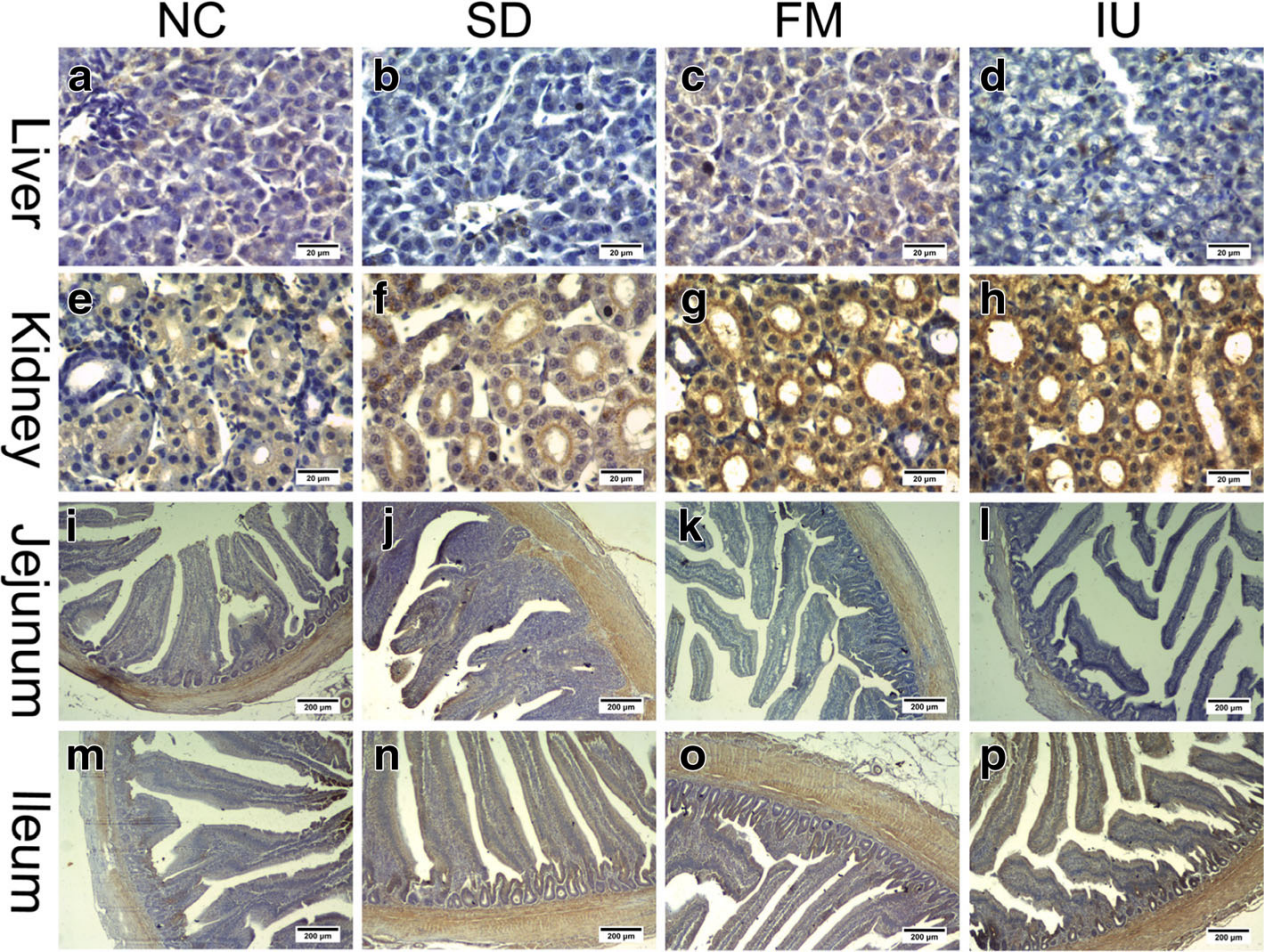
BCRP protein expression in the liver, kidney, jejunum, and ileum of chickens in different treatment groups based on immunohistochemistry. NC: control group; SD: sulfonamide-supplemented group; FM: fish meal group; IU: injection uric acid group. **a** to **h**: scale bar = 20 μm; I to P: scale bar = 200 μm

**Fig. 8:**
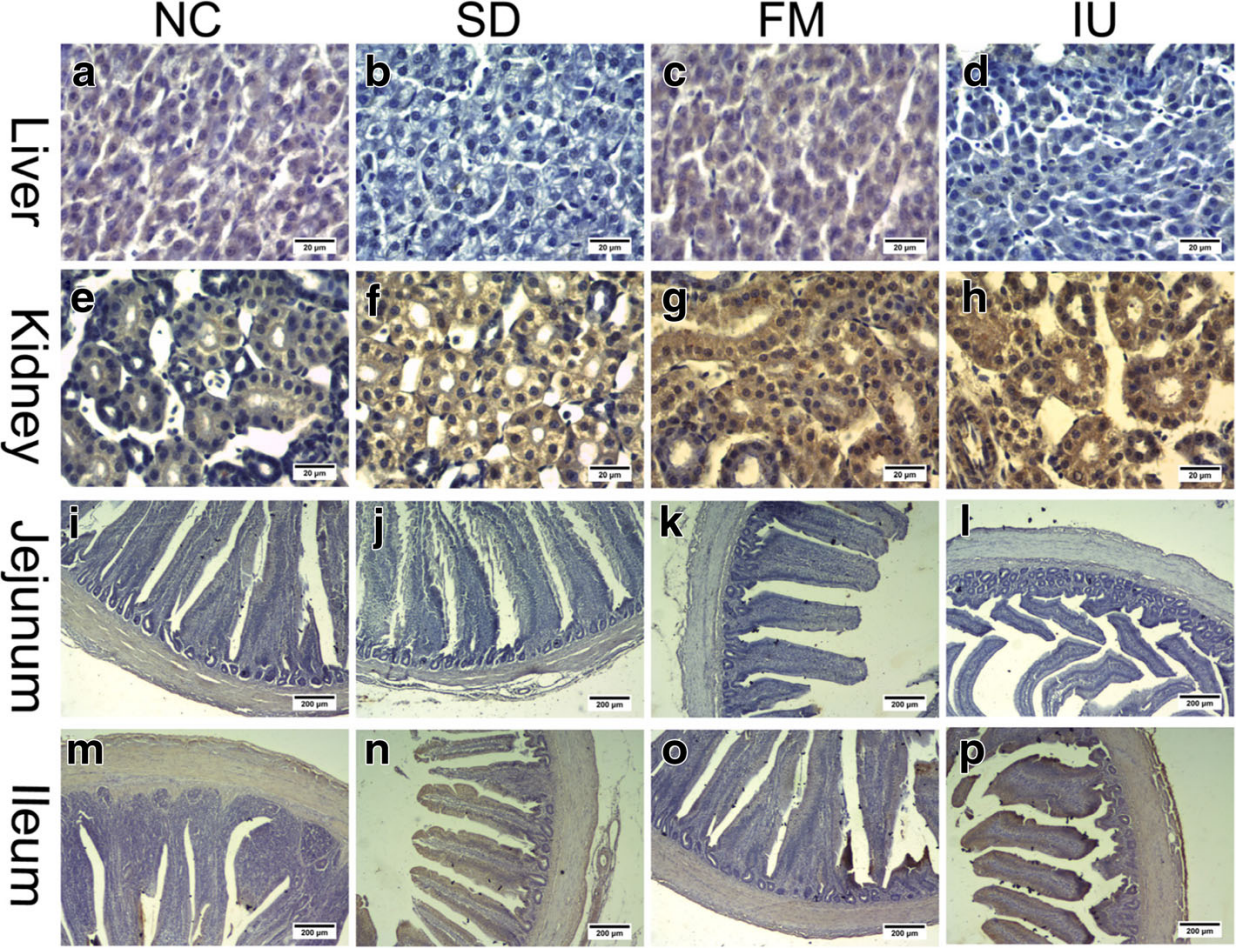
MRP4 protein expression in the liver, kidney, jejunum, and ileum of chickens in different treatment groups based on immunohistochemistry. NC: control group; SD: sulfonamide-supplemented group; FM: fish meal group; IU: injection uric acid group. **a** to **h**: scale bar = 20 μm; I to P: scale bar = 200 μm

Finally, we used immunohistochemistry to evaluate BCRP and MRP4 protein expression levels in the liver, kidney, jejunum, and ileum in each group. As shown in Fig. 7, Fig. 8, and Table 3, liver BCRP and MRP4 protein expression levels were lower in the SD and IU groups than in the NC group, with the decrease in the SD group being significant (*P* < 0.05). In the FM group, liver expression levels were similar to those in the NC group. Renal and ileal BCRP and MRP4 expression levels were significantly increased (*P* < 0.01) in all experimental groups, except in the SD group, where renal MRP4 expression was slightly increased, whereas jejunum BCRP and MRP4 levels were lower than those in the NC group.

**Table 3:** Average optical density values of BCRP and MRP4 in the liver, kidney, jejunum, and ileum in all treatment groups based on immunohistochemistry

Protein	Item	NC	SD	FM	IU
BCRP	Liver	0.039 ± 0.0032	0.027 ± 0.0058	0.045 ± 0.0046	0.027 ± 0.003
	kidney	0.081 ± 0.0031	0.125 ± 0.0081^**^	0.198 ± 0.0128^**^	0.211 ± 0.0061^**^
	Jejunum	0.067 ± 0.0034	0.064 ± 0.0022	0.054 ± 0.0036	0.050 ± 0.0158
	Ileum	0.062 ± 0.0078	0.131 ± 0.0021^**^	0.083 ± 0.0028^*^	0.197 ± 0.0067^**^
MRP4	Liver	0.035 ± 0.0017	0.022 ± 0.0016^**^	0.040 ± 0.0039	0.029 ± 0.0023
	kidney	0.097 ± 0.0043	0.115 ± 0.0092	0.165 ± 0.0007^**^	0.202 ± 0.0074^**^
	Jejunum	0.064 ± 0.0025	0.052 ± 0.0020	0.048 ± 0.0074	0.045 ± 0.0079
	Ileum	0.060 ± 0.0046	0.081 ± 0.0113	0.068 ± 0.0056	0.125 ± 0.0047^**^

^*^P < 0.05, ^**^P < 0.01 compared with the NC group

*NC*: control group; *SD*: sulfonamide-supplemented group; *FM*: fish meal group; *IU*: injection uric acid group. All data are means ± SE; *N* = 9 samples per treatment

### Correlation between *BCRP* and *MRP4* mRNA expression

**Fig. 9:**
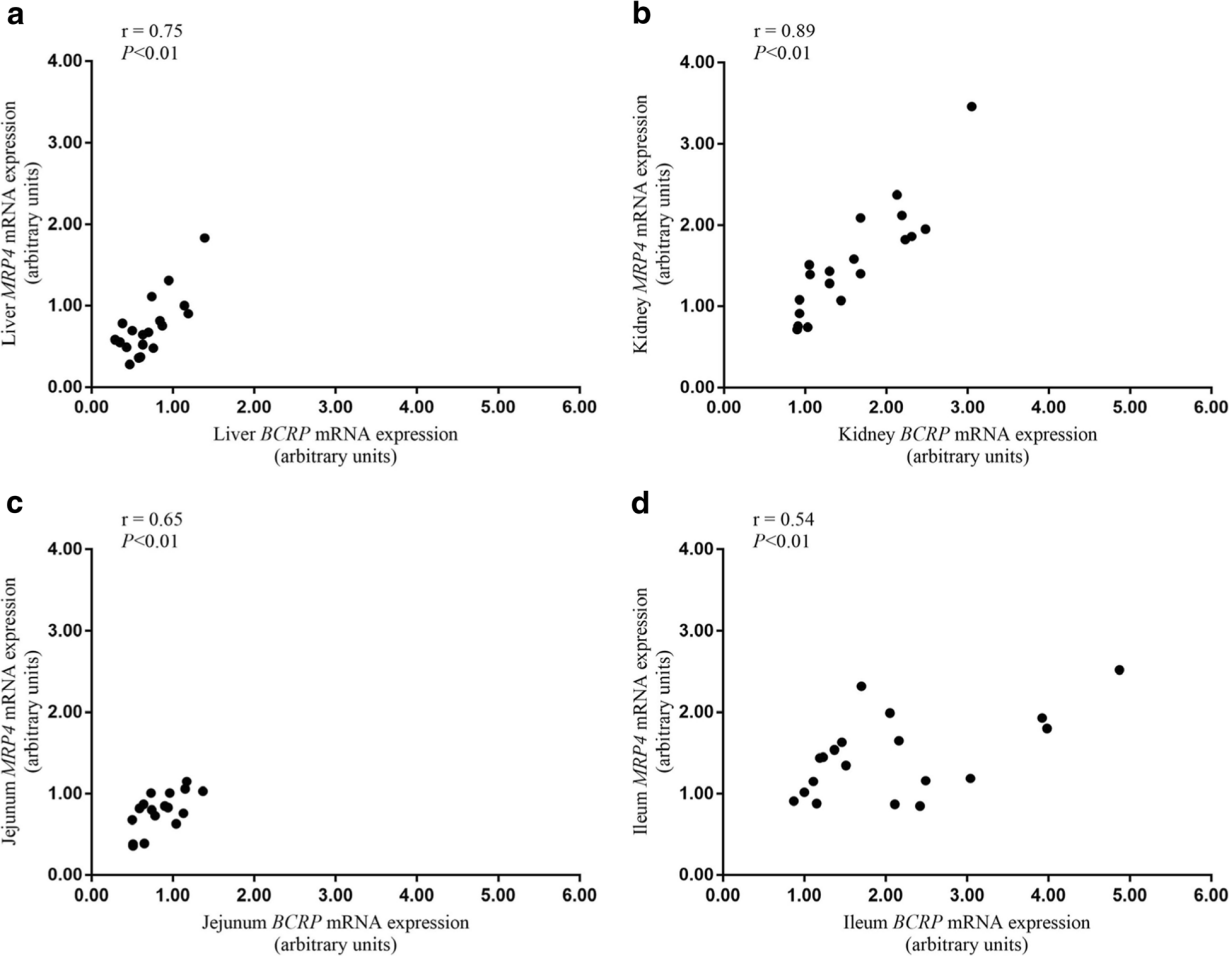
Correlation between *BCRP* and *MRP4* mRNA expression in the liver, kidney, jejunum, and ileum of chickens. The data of this scatter plots and correlation analysis are from QPCR. And the correlation analysis was conducted by Pearson’s correlation coefficients. **a**: Liver, **b**: Kidney, **c**: Jejunum, **d**: Ileum

To determine the relationship between *BCRP* and *MRP4* expression, *BCRP* and *MRP4* mRNA expression levels of each sample were used for correlation analysis. As shown in Fig. 9, *BCRP* mRNA expression was positively correlated with *MRP4* mRNA expression in the liver, kidney, jejunum, and ileum (*P* < 0.01).

## Discussion

BCRP and MRP4 are uric acid transporters present in various organs, such as the human liver, kidney, and intestines, and can be expressed in heterogeeous systems for uric acid transport [10, 12, 33]. BCRP and MRP4 are involved in human and mouse uric acid excretion, and in-vitro experiments have revealed that BCRP and MRP4 are expressed in cPTCs [13]. However, their roles in the chicken uric acid transport system remain unclear. The results of this study showed that BCRP and MRP4 are highly expressed in the jejunum and ileum of chickens, with low expression in the liver and kidney and minimal expression of BCRP in the kidney. Several BCRP localization studies have reported relatively high expression in rat and mouse kidney as well as in the small intestine, especially in the ileum [34], whereas in humans, the apical membrane of hepatocytes, colonic epithelial cells, and placental syncytium trophoblasts exhibit relatively high expression [35, 36]. In humans, MRP4 is most strongly expressed in the kidney, followed by the liver and intestines [37]. However, in mice, MRP4 levels are significantly higher in the kidney than in the liver and intestines, and liver and kidney expression levels are significantly higher in female than in male mice [38]. These results indicate that although BCRP and MRP4 can be expressed heterologously, their tissue distributions differ among species and these differences may be related to species-specific mechanisms of uric acid metabolism. Previous studies have shown that endogenous uric acid in mice is secreted directly from the blood into the intestinal lumen of all bowel segments [17, 39]. Ileal secretion is approximately 3-fold and 2-fold higher than jejunal and colonic secretion, respectively [16]. These findings indicate that the ileum is the main site of intestinal uric acid secretion in mice [39]. The results of this study demonstrated that in chickens, BCRP and MRP4 are mainly expressed in the jejunum and ileum, with higher expression in the ileum; accordingly, the roles of BCRP and MRP4 in ileal uric acid excretion may be particularly important.

The kidney are recognized as the main regulators of serum uric acid, and the excretion of renal uric acid is determined by the balance between uric acid reabsorption and re-secretion [40]. In humans, approximately 70% of uric acid is secreted into the urine through the renal tubules [7]. BCRP and MRP4 are critical for uric acid secretion in human and mouse kidney [41]. A high-protein diet can increase chicken serum uric acid levels [42]. However, in this study, the high-protein diet FM group did not demonstrate an increase in serum uric acid, creatinine, or BUN levels, whereas renal BCRP and MRP4 expression increased significantly and ileal expression increased slightly. These findings indicate that when renal function is normal, the kidney are the main site of uric acid clearance and that renal BCRP and MRP4 are involved in renal uric acid excretion.

In this study, serum uric acid, creatinine, and BUN levels were significantly increased in the SD and IU groups compared with those in the NC group, and renal tubular epithelial cells were damaged. In the SD group, sulfonamide crystallization may have blocked the renal tubules and caused renal damage [43], thereby reducing uric acid excretion and increasing serum uric acid. In the IU group, intraperitoneal injection of uric acid not only raised the serum uric acid levels, but also caused renal damage [44, 45]. Mouse studies have shown that the ileum plays an important role in ileal uric acid clearance during kidney injury [18, 19]. Similarly, our results showed that chicken serum uric acid increased when serum creatinine and BUN levels were elevated in the SD and IU groups. In addition, BCRP and MRP4 protein and gene expression levels in the ileum were significantly increased. These results suggest that kidney and intestinal BCRP and MRP4 are involved in chicken uric acid clearance and that when renal function is impaired, uric acid excretion in the ileum can provide a compensatory mechanism by increasing BCRP and MRP4 expression. In addition, BCRP and MRP4 levels in the jejunum were slightly lower in the three experimental groups than in the NC group. The mechanisms underlying these differences remain to be evaluated in future studies.

Serum uric acid levels in the SD and IU groups were significantly higher than those in the NC group, whereas liver BCRP and MRP4 expression levels were significantly lower. In the FM group, serum uric acid levels were not altered, and liver BCRP and MRP4 expression levels were slightly increased. These results indicate that changes in liver BCRP and MRP4 expression are inversely correlated with changes in serum uric acid levels. Previous studies have shown that BCRP and MRP4 are expressed as uric acid efflux proteins in the basolateral membrane of hepatocytes [46]. The findings in this study indicated that BCRP and MRP4 may participate in liver uric acid entry into the blood circulation. The decrease in liver BCRP and MRP4 expression with increasing serum uric acid level may be a mechanism to reduce the serum uric acid level.

This study had some limitations. The mechanisms underlying the relationship between changes in serum uric acid levels and liver, kidney, and intestinal BCRP and MRP4 levels remain unclear. Although *BCRP* is positively related to *MRP4* mRNA expression, the potential interaction between BCRP and MRP4 remains to be elucidated. In addition, this study considered only two transporters and thus other transporters involved in chicken uric acid excretion should be studied. In addition, the expression of BCRP and MRP4 in other tissues needs to be studied in the future.

## Conclusions

Our results show that BCRP and MRP4 participate in renal and intestinal uric acid excretion in chickens and that *BCRP* is positively related to *MRP4* mRNA expression. When renal function is impaired, BCRP and MRP4 expression in the ileum exhibit compensatory increases; however, when renal function is normal, renal BCRP and MRP4 are the main regulators of uric acid excretion with ileal BCRP and MRP4 expression having no significant influence. Mechanistic insights into the roles of BCRP and MRP4 in chicken uric acid secretion remain to be investigated in future studies.

## Abbreviations

BCRP: Breast cancer resistance protein
BUN: Blood urea nitrogen
cPTCs: Chicken renal proximal tubular epithelial cells
IOD: Integrated optical density
MRP4: Multidrug resistance protein 4
QPCR: Quantitative Real-time PCR
TEM: Transmission electron microscopy
